# Irisin and the Metabolic Phenotype of Adults with Prader-Willi Syndrome

**DOI:** 10.1371/journal.pone.0136864

**Published:** 2015-09-03

**Authors:** Harry J. Hirsch, Itai Gross, Yehuda Pollak, Talia Eldar-Geva, Varda Gross-Tsur

**Affiliations:** 1 Israel Multidisciplinary Prader-Willi Syndrome Clinic, Neuropediatric Unit, Department of Pediatrics, Shaare Zedek Medical Center, Jerusalem, Israel; 2 Department of Pediatrics, Hadassah Hospital, Ein Kerem, Jerusalem, Israel; 3 The School of Education, The Hebrew University, Jerusalem, Israel; 4 The Hebrew University Faculty of Medicine, Jerusalem, Israel; 5 Reproductive Endocrinology and Genetics Unit, Department of Obstetrics and Gynecology, Shaare Zedek Medical Center, Jerusalem, Israel; Monash University, AUSTRALIA

## Abstract

**Context:**

Hyperphagia, low resting energy expenditure, and abnormal body composition contribute to severe obesity in Prader Willi syndrome (PWS). Irisin, a circulating myokine, stimulates “browning” of white adipose tissue resulting in increased energy expenditure and improved insulin sensitivity. Irisin has not been previously studied in PWS.

**Objectives:**

Compare plasma and salivary irisin in PWS adults and normal controls. Examine the relationship of irisin to insulin sensitivity and plasma lipids.

**Design and Study Participants:**

A fasting blood sample for glucose, lipids, insulin, leptin, adinopectin, and irisin was obtained from 22 PWS adults and 54 healthy BMI-matched volunteers. Saliva was collected for irisin assay in PWS and controls.

**Results:**

Fasting glucose (77±9 vs 83±7mg/dl, p = 0.004), insulin (4.1±2.0 vs 7.9±4.7μU/ml, p<0.001), and triglycerides (74±34 vs 109±71mg/dl, p = 0.007) were lower in PWS than in controls. Insulin resistance (HOMA-IR) was lower (0.79±0.041 vs 1.63±1.02, p<0.001) and insulin sensitivity (QUICKI) was higher (0.41±0.04 vs 0.36±0.03, p<0.001) in PWS. Plasma irisin was similar in both groups, but salivary irisin (64.5±52.0 vs 33.0±12.1ng/ml), plasma leptin (33.5±24.2 vs 19.7±19.3ng/ml) and plasma adinopectin (13.0±10.8 vs 7.6±4.5μg/ml) were significantly greater in PWS (p<0.001). In PWS, plasma irisin showed positive Pearson correlations with total cholesterol (r = 0.58, p = 0.005), LDL-cholesterol (r = 0.59, p = 0.004), and leptin (r = 0.43, p = 0.045). Salivary irisin correlated negatively with HDL-cholesterol (r = -0.50, p = 0.043) and positively with LDL-cholesterol (r = 0.51, p = 0.037) and triglycerides (r = 0.50, p = 0.041).

**Conclusions:**

Salivary irisin was markedly elevated in PWS although plasma irisin was similar to levels in controls. Significant associations with plasma lipids suggest that irisin may contribute to the metabolic phenotype of PWS.

## Introduction

Prader—Willi syndrome (PWS) is a complex neurogenetic disorder resulting from the absence of paternal expression of imprinted genes in the 15q11–q13 region. Clinical features include morbid obesity, cognitive disabilities, behavioral disorders, short stature, hypogonadism, and autonomic dysregulation, e.g. alterations in temperature regulation. PWS individuals lack a sense of satiety, resulting in obsessive craving for food. Severe restriction of caloric intake is needed to prevent complications of obesity, such as obstructive sleep apnea, pulmonary hypertension, and type 2 diabetes mellitus [1].

The metabolic profile of individuals with PWS is unique. Compared to BMI- matched controls, fasting insulin levels are lower and insulin sensitivity is increased [2–5]. Resting energy expenditure is markedly decreased due to decreased activity and decreased lean body mass [6]. Visceral adipose tissue is reduced in PWS relative to non-PWS obese controls [7,8].

White adipose tissue (WAT) is a major source of energy storage. WAT cells contain few mitochondria and show relatively little metabolic activity. Brown adipose tissue (BAT) found mainly in infants and young children is also present in many adults [9]. In contrast to WAT, BAT dissipates energy by activating uncoupling protein-1 (UCP-1) which by-passes ATP production in the inner mitochondrial membrane of BAT cells, thereby releasing energy in the form of heat. BAT contributes to increased insulin sensitivity and decreases susceptibility to weight gain [10].

Exercise stimulates cells within subcutaneous WAT to acquire certain characteristics of BAT, namely increased numbers of mitochondria, and expression of the uncoupling protein. Studies in mice have shown that stimulation of this “beige” or “brite” adipose tissue from subcutaneous WAT, results in increased insulin sensitivity, improvement in glucose tolerance, and decreased weight gain [11]. Bostrom et al identified a peptide molecule, irisin, the circulating cleavage product of skeletal muscle fibronectin type III domain-containing protein 5 (FNDC5) which promotes subcutaneous WAT to express BAT-like properties by inducing UCP-1 [10]. A modest increase in circulating irisin levels in mice fed a high-fat diet resulted in lower body weight, prevented diet-induced insulin resistance, improved glucose tolerance, and prolonged survival [10,11].

Assessment of quantity or activity of BAT in PWS might contribute to understanding some aspects of the unique metabolic phenotype in this syndrome, however, *in vivo* assessment of BAT requires performing a combined PET/CT scan. For ethical reasons, it is not feasible to perform this test which involves radiation exposure on PWS individuals for purposes of research investigation. With increasing evidence that irisin may promote “browning” of white adipocytes, we chose to investigate the association of plasma irisin with metabolic parameters and adipokines in PWS individuals. Since skeletal muscle is the main source of circulating irisin, we hypothesized that PWS individuals, in whom lean body mass is known to be decreased, might have lower serum irisin levels compared to obese and lean controls. We sought to determine if irisin contributes to the unique metabolic profile in PWS.

## Study Participants and Methods

### Patients and volunteers

Twenty-two individuals with PWS (12 males/10 females) ages 18.0–39.3 years, and mean body mass index (BMI) 29.2±8.6 kg/m^2^ (range 22.1–59.1), who live in residential homes for PWS, were recruited from the Israeli National Multidisciplinary PWS Clinic at Shaare Zedek Medical Center, Jerusalem, Israel. The diagnosis was confirmed by molecular genetic studies of chromosome 15 including microdeletion in 11 (7M/4F) and uniparental disomy (UPD) in 11 (5M/6F). All PWS patients received combinations of atypical anti-psychotics, benzodiazepines, dopamine antagonists, SSRI (selective serotonin reuptake inhibitors), and anticonvulsant medications. None were receiving growth hormone at the time of the study. The control group was comprised of 29 individuals (14 males/15 females) with BMI ˂ 25 kg/m^2^ (21.8±2.0, range 18.8–24.8) ages 19–40.6 years and 25 overweight or obese volunteers (12 males/13 females) with BMI ˃ 25 kg/m^2^ (30.3±5.7, range 25.1–45.7) ages 18.6–39.8. Exclusion criteria included chronic illnesses, uncontrolled hypothyroidism, diabetes mellitus, or exposure to hormone medication including estrogen-containing compounds, glucocorticoids, or androgens. The study was approved by the institutional review board of Shaare Zedek Medical Center. Written informed consent was obtained from the patients and/or legal guardians as well as from the overweight and normal-BMI volunteers.

### Methods

Height was measured with a wall-mounted stadiometer. Weight (in light clothing) and waist and hip circumferences were measured and recorded.

Fasting blood samples were taken after an overnight fast between 0800 and 1000 for blood glucose, cholesterol, HDL-cholesterol, LDL-cholesterol, triglycerides, hemoglobin A1c, CRP, insulin, irisin, leptin, and adinopectin. Aprotinin (Trasylol) (500 kIU/ml of blood) was added to each EDTA vacutainer tube. Blood samples were centrifuged at 4000 rpm for 10 min and the plasma was removed and stored at -70°C until time of the assay. Saliva samples were collected using Salivettes (Sarstedt, Nurnbrecht, Germany). Saliva samples were centrifuged for 15 min at 4000 rpm to remove any particles or sediments. Aprotinin (500 kIU/ml of saliva) was added to each collection tube and samples were stored at −70°C prior to assay.

### Assays

Plasma glucose, cholesterol, HDL-cholesterol, and triglycerides were assayed using a Vitros automated analyser, Ortho Clinical Diagnostics, Johnson and Johnson. Inter- and intra-assay variation were < 3%. Hemoglobin A1c was measured by capillary electrophoresis (Capillarys 2, Sebia) with an inter- and intra-assay coefficient of variation of <2.5%. C-reactive protein (CRP) was measured with a Berhing Nephelometer Analyser II (Siemens). Plasma insulin was measured using an Architect system, Abbot Laboratories, USA. The lower limit of sensitivity was 1.0 μU/ml; intra- and inter-assay coefficients of variation were less than 7%.

Measurement of leptin, adinopectin, and irisin (catalogue number EK-067-29) were performed by ELISA (Phoenix, Pharmaceuticals, USA). Assay of plasma samples for each respective peptide were performed in one run, thereby avoiding inter-assay variation. Samples for salivary irisin were assayed in a separate run. Plasma and saliva samples were diluted with 1:41 with buffer according to the manufacturer’s recommendations. Lower limits of detection for leptin, adinopectin, and irisin were 0.312 ng/ml, 0.15 ng/ml, and 1.3 ng/ml, respectively. Intra-assay variations were <10%. Inter-assay variation for irisin, according to the manufacturer, was <15%.

HOMA-IR (homeostatic model assessment of insulin resistance), a measure of insulin resistance, was calculated according to the standard formula [12] “[glucose (mg/dl) x insulin (μU/ml)]/405.” QUICKI (quantitative insulin sensitivity check index), a measure of insulin sensitivity, was calculated according to the formula “1 / [log(fasting insulin in μU/ml) + log(fasting glucose in mg/dl)]”[13]. HOMA-beta (homeostatic model assessment of β-cell function), a measure of beta-cell function was calculated according to the formula [14] “360 x insulin in μU/ml)/(glucose in mg/dl– 63).”

### Statistical analysis

Samples with hormone values below the assay detection limit were assigned the detection limit value. Male:female ratios among groups were analysed using the Chi-square test. The distribution of each clinical and laboratory test variable was examined by the Kolomogorov-Smirnov test. Since almost all of the variables were normally distributed, further analyses were conducted using parametric tests. Differences in clinical and hormone variables among the four groups (obese/overweight PWS, lean PWS, obese/overweight controls, and lean controls) were analysed using a 2 by 2 ANOVA, followed by post-hoc Tukey tests, comparing each pair of groups. CRP levels were not normally distributed and were further analysed using Kruskal-Wallis and Mann-Whitney tests. The Pearson test was used to assess correlations among blood tests and clinical variables. A p value of less than 0.05 was considered to be significant.

## Results

### Clinical features

As shown in table 1, the PWS and control groups were similar with respect to male:female ratio, age, BMI, and waist/hip ratio. In table 2, results for 14 PWS patients with BMI >25kg/m^2^ (“overweight”) are compared with those of 25 normal volunteers with BMI >25kg/m^2^. Data for the remaining 8 PWS patients whose BMI is <25kg/m^2^ are compared with results of 29 normal volunteers with BMI <25kg/m^2^. There were no differences between each of BMI-matched PWS and control groups with regard to sex ratio, age, or waist-hip ratio.

**Table 1:** Clinical and biochemical characteristics in PWS and normal controls.

Group:	PWS	Controls	P value PWS vs. Healthy
n	22	54	
Males/females	12/10	26/28	0.501
Age (years)	28.7±5.9	28.3±6.2	0.986
DEL/UPD	11/11		
BMI (kg/m^2^)	29.2±8.6	25.7±5.9	0.186
Waist/hip ratio	0.91±0.09	0.87±0.07	0.18
CRP	0.527	0.439	0.051
Glucose (mg/dl)	**77±9**	**83±7**	**0.004**
HbA1c (%)	5.1±0.4	5.3±0.3	0.108
Total cholesterol (mg/dl)	156±34	162±28	0.216
LDL-cholesterol (mg/dl)	95±30	91±23	0.94
HDL-cholesterol (mg/dl)	46±12	49±13	0.618
Triglycerides (mg/dl)	**74±34**	**109±71**	**0.007**
Insulin (μU/ml)	**4.1±2.0**	**7.9±4.7**	**<0.001**
HOMA-IR	**0.79±0.41**	**1.63±1.02**	**<0.001**
HOMA-β	115.9±68.9	159.0±100.9	0.055
QUICKI	**0.41±0.04**	**0.36±0.03**	**<0.001**
Leptin (ng/ml)	**33.5±24.2**	**19.7±19.3**	**<0.001**
Adinopectin (μg/ml)	**13.0±10.8**	**7.6±4.5**	**<0.001**
Plasma irisin (ng/ml)	58.2±5.1	57.1±8.6	0.704
Salivary irisin (ng/ml)	**64.5±52.0** (n = 17)	**33.0±12.1**	**<0.001**

Mean±SD values for clinical and laboratory parameters for PWS patients and non-PWS controls. Values which are significantly different between the two groups appear in bold. Differences between the two were analysed using a 2 by 2 ANOVA. CRP levels were not normally distributed and analysed using Mann-Whitney tests. Values which differ significantly between the groups are shown in bold font.

**Table 2:** Clinical and biochemical characteristics in PWS and normal controls according to BMI status (obese/overweight vs normal BMI).

Group:	PWS Overweight and obese	Controls Overweight and obese	P value PWS Ob vs Control Ob	PWS Normal BMI	Controls Normal BMI	P value PWS lean vs Control lean
n	14	25		8	29	
Males/females	8/6	12/13	0.462	4/4	14/15	0.931
Age (years)	29.8±4.8	29.3±6.7	0.994	26.9±7.4	27.4±5.7	0.996
DEL/UPD	5/9			6/2		
BMI (kg/m^2^)	33.0±8.8	30.3±5.7	0.503	22.7±1.4	21.8±2.0	0.974
Waist/hip ratio	0.93±0.10	0.90±0.07	0.375	0.86±0.06	0.85±0.06	0.978
CRP	0.60±0.49	0.53±0.60	0.95	0.39±0.11	0.36±0.06	0.997
Glucose (mg/dl)	**77±11**	**85±8**	**0.013**	77±3	80±5	0.587
HbA1c (%)	5.1±0.4	5.3±0.4	0.637	5.1±0.2	5.2±0.3	0.672
Total cholesterol (mg/dl)	165±38	168±30	0.535	141±17	156±26	0.535
LDL-cholesterol (mg/dl)	105±33	95±23	0.598	79±15	88±24	0.781
HDL-cholesterol (mg/dl)	42±8	45±9	0.905	52±16	52±15	1.000
Triglycerides (mg/dl)	**87±37**	**141±87**	**0.03**	52±7	82±38	0.576
Insulin (μU/ml)	**4.5±2.2**	**9.7±6.0**	**0.001**	3.6±1.6	6.4±2.8	0.299
HOMA-IR	**0.86±0.46**	**2.04±1.26**	**<0.001**	0.68±0.30	1.28±0.59	0.282
HOMA-β	125.7±75.1	180.3±128.4	0.319	99.9±58.7	140.6±66.3	0.692
QUICKI	**0.41±0.04**	**0.35±0.02**	**<0.001**	**0.42±0.03**	**0.38±0.03**	**0.004**
Leptin (ng/ml)	39.8±25.1	30.1±21.7	0.581	22.5±19.0	10.7±11.0	0.41
Adinopectin (μg/ml)	9.62±6.78	6.71±3.28	0.368	**19.1±14.1**	**8.3±5.2**	**0.001**
Plasma irisin (ng/ml)	58.8±6.1	58.2±8.9	0.991	57.1±2.4	56.1±8.4	0.99
Salivary irisin (ng/ml)	**81.6±67.2** (n = 9)	**34.1±13.3**	**<0.001**	42.7±20.5	32.0±11.0	0.739

Mean±SD values for clinical and laboratory parameters for overweight PWS patients (BMI > 25 kg/m^2^) compared with overweight control volunteers, anda for PWS patients whose BMI was less than 25 kg/m^2^ compared with lean control participants. Values which are significantly different between the two groups appear in bold. Differences among the four groups were analysed using a 2 by 2 ANOVA, followed by post-hoc Tukey tests. CRP levels were not normally distributed and were analysed using Kruskal-Wallis and Mann-Whitney tests. Values which differ significantly between the groups are shown in bold font.

### Glucose, lipids, insulin, and measures of insulin sensitivity

Although CRP was slightly increased in PWS vs controls (Table 1), the difference did not achieve statistical significance (p = 0.051). Fasting glucose was slightly lower in PWS compared to healthy controls (77±9 vs 83±7 mg/dl, p = 0.004) despite similar BMI values for each group. Hemoglobin A1c values, total cholesterol, LDL- and HDL-cholesterol levels did not differ between the PWS and control groups, but triglycerides were lower in PWS (74±34 vs 109±71 mg/dl, p = 0.007). While fasting glucose was only slightly lower in PWS, mean fasting insulin was nearly 50% lower compared to BMI-matched controls (4.1±2.0 vs 7.9±4.7 μU/ml, p<0.001). Insulin resistance as measured by HOMA was less (0.79±0.41 vs 1.63±1.02, p<0.001) and insulin sensitivity measured by QUICKI was increased (0.41±0.04 vs 0.36±0.03, p<0.001) in PWS compared to healthy controls. No significant difference in beta-cell function as assessed by HOMA-beta was seen in PWS vs controls.

In table 2, results for the 14 overweight/obese PWS patients are shown separately from those of the 8 PWS patients with normal BMI. Comparing the overweight PWS patients with overweight controls, significant differences were found for fasting glucose (77±11 vs 85±8 mg/dl, p = 0.013) and triglycerides (87±37 vs 141±87 mg/dl, p<0.001). HbA1c, total cholesterol, LDL-cholesterol, and HDL-cholesterol did not differ between the two groups. Fasting insulin was markedly lower in the overweight PWS group compared to overweight controls (4.5±2.2 vs 9.7±6.0 μU/ml, p = 0.001). Overweight PWS patients had less insulin resistance and greater insulin sensitivity than was found in BMI-matched controls (HOMA: 0.86±0.46 vs 2.04±1.26, p<0.001; QUICKI: 0.41±0.04 vs0.35±0.02, p<0.001). HOMA-beta was similar in PWS and controls for the respective BMI groups.

Results for PWS patients with normal BMI (Table 2) showed no significant differences in levels of glucose, cholesterol, HDL-cholesterol, LDL-cholesterol, or triglycerides compared with healthy controls of comparable BMI. Fasting insulin and HOMA-IR did not significantly differ between the two groups with normal BMI, although insulin sensitivity (QUICKI) was slightly, but significantly higher in the PWS group (0.42±0.03 vs 0.38±0.03, p = 0.004).

### Leptin and adinopectin

Plasma leptin concentrations were significantly greater in the entire PWS cohort compared to controls (33.5±24 vs.19.7±19.3 ng/ml, p<0.001) (Table 1). Leptin in overweight PWS patients did not, however, differ from levels in the overweight controls and levels in PWS patients with normal BMI did not differ from levels in the normal-BMI control group (table 2). Adinopectin levels in PWS were higher than levels found in the control group (13.0±10.8 μg/ml vs, 7.6±4.5 μg/ml, p<0.001). When the overweight PWS group was compared with overweight controls, there was no significant difference in adinopectin. PWS adults with normal BMI did, however, have higher plasma adinopectin compared to BMI-matched controls (19.1±14.1 vs. 8.3±5.2 μg/ml, p = 0.001) as shown in Table 2.

### Plasma and salivary irisin

Plasma irisin concentrations were similar in PWS patients and controls (58.2±5.1 vs. 57.1±8.6 ng/ml, NS) (Table 1). No differences in plasma irisin were seen in males vs females for PWS patients or controls. Salivary irisin concentrations, however, were twice as high in PWS than in the control group (64.5±52.0 vs. 33.0±12.1 ng/ml, p<0.001). Salivary irisin concentrations (Table 2) were highest in the overweight PWS patients and were significantly greater than levels found in the overweight controls (81.6±67.2 vs 34.1±13.3, p<0.001). No sex differences were seen in salivary irisin for PWS patients or controls. Due to difficulties in patient cooperation, it was not possible to obtain a saliva sample from five PWS patients whose BMI was greater than 25 kg/m^2^ (Tables 1 and 2). In PWS patients with normal BMI, salivary irisin did not differ from concentrations seen in BMI-matched controls.

Plasma irisin in PWS adults correlated with cholesterol (r = 0.58, p = 0.005), LDL-cholesterol (r = 0.59, p = 0.004), and leptin (r = 0.43, p = 0.045) (Table 3). In the non-PWS controls, plasma irisin correlated with waist/hip ratio (r = 0.34, p = 0.013), HOMA-beta (r = 0.28, p = 0.04), total cholesterol (r = 0.42, p = 0.002), and LDL-cholesterol (r = 0.49, p = 0.002).

**Table 3:** Pearson correlations of plasma irisin with clinical and biochemical parameters.

	PWS (n = 22)	P value	Controls (n = 54)	P value
BMI (kg/m^2^)	0.13	0.517	0.16	0.251
Waist/hip ratio	-0.14	0.884	**0.34**	**0.013**
Glucose (mg/dl)	0.16	0.474	-0.04	0.787
Insulin (μIU/ml)	-0.10	0.651	0.25	0.065
Hemoglobin A1c (%)	0.18	0.457	0.12	0.386
HOMA-IR	-0.05	0.823	0.25	0.068
HOMA-β	0.17	0.943	**0.28**	**0.040**
QUICKI	0.04	0.866	-1.61	0.244
Cholesterol (mg/dl)	**0.58**	**0.005**	**0.42**	**0.002**
HDL-cholesterol (mg/dl)	0.04	0.856	-0.27	0.052
LDL-cholesterol (mg/dl)	**0.59**	**0.004**	**0.49**	**<0.001**
Triglycerides (mg/dl)	0.16	0.470	0.25	0.071
Leptin (ng/ml)	**0.43**	**0.045**	0.06	0.670
Adinopectin (ng/ml)	-0.25	0.271	-0.11	0.415
Salivary irisin (ng/ml)	0.44	0.078	**0.31**	**0.024**

Pearson correlations of plasma irisin with clinical and laboratory parameters in PWS patients and non-PWS controls. Values for which the correlations are statistically significant (p < 0.05) are shown in bold font.

**Fig 1:**
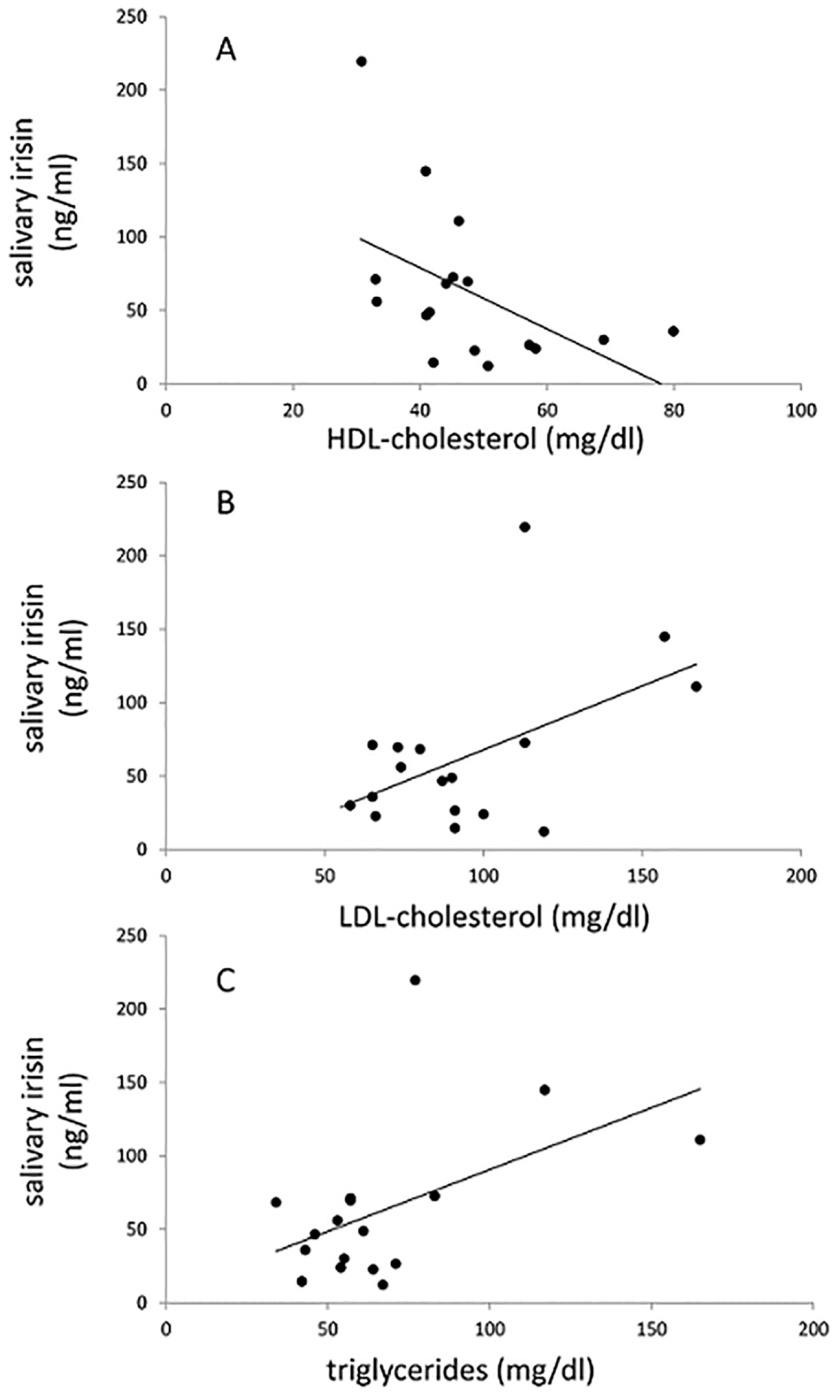
Correlations between salivary irisin and plasma HDL-cholesterol (A), LDL-cholesterol (B), and triglycerides (C). Pearson correlations and p values are shown in Table 4.

There was a significant correlation between plasma irisin and salivary irisin for the control group (r = 0.31, p = 0.024) but not for the PWS patients. Salivary irisin in PWS patients correlated inversely with HDL-cholesterol (r = −0.50, p = 0.043) and positively with LDL-cholesterol (r = 0.51, p = 0.037) and triglycerides (r = 0.50, p = 0.041) (Fig 1 and Table 4). In the control group, salivary irisin correlated only with plasma irisin, but not with any other parameters.

**Table 4:** Pearson correlations of salivary irisin with clinical and biochemical parameters.

	PWS (n = 17)	P value	Controls (n = 54)	P value
BMI (kg/m^2^)	0.17	0.517	0.02	0.911
Waist/hip ratio	0.04	0.884	-0.16	0.253
Glucose (mg/dl)	0.46	0.065	0.16	0.236
Insulin (μIU/ml)	0.13	0.614	0.05	0.727
Hemoglobin A1c (%)	-0.42	0.137	-0.04	0.758
HOMA-IR	0.27	0.291	0.09	0.524
HOMA-β	-0.124	0.648	-0.10	0.470
QUICKI	-0.25	0.334	-0.01	0.935
Cholesterol (mg/dl)	0.35	0.163	0.10	0.457
HDL-cholesterol (mg/dl)	**-0.50**	0.043	-0.04	0.801
LDL-cholesterol (mg/dl)	**0.51**	0.037	0.17	0.226
Triglycerides (mg/dl)	**0.50**	0.041	-0.35	0.801
Leptin (ng/ml)	0.15	0.569	0.10	0.466
Adinopectin (ng/ml)	-0.32	0.205	0.10	0.478

Pearson correlations of salivary irisin with clinical and laboratory parameters in PWS patients and non-PWS controls. Values for which the correlations are statistically significant (p < 0.05) are shown in bold font.

## Discussion

This study is the first report of irisin in Prader-Willi syndrome. We found that irisin concentrations in saliva from PWS adults were markedly elevated compared to levels in control volunteers, although plasma irisin did not differ between PWS and controls. Previous studies demonstrated higher concentrations of irisin in saliva compared to plasma [15,16]. We anticipated that salivary irisin might reveal significant differences between the PWS and control population which might not be apparent from the lower levels found in plasma.

Plasma irisin correlated with salivary insulin in normal controls but not in PWS suggesting that the regulation of irisin production and/or metabolism may differ in these two populations.

Although saliva in PWS is characteristically thicker with higher protein output than in normal controls it is not likely that the elevated irisin levels are due to the abnormal saliva [17,18]. We measured sodium and protein concentrations in saliva supernatant from five PWS individuals and five control volunteers [S2 Dataset. Saliva sodium and protein concentrations in PWS and controls]. PWS saliva had higher concentrations of sodium (33±23 vs 19±8 mEq/l, p = 0.25) and protein (0.88±41 vs 0.51±21 mg/ml, p = 0.11) compared to controls. All salivary samples were centrifuged immediately after collection and the samples were diluted 1:41 with buffer prior to assay. After dilution, the differences in sodium and protein concentrations were 0.34 mEq/l and 0.009mg/ml, respectively. It is unlikely that such small differences in saliva composition would account for the increased immunoreactive irisin levels which we found in our PWS patients. Furthermore, the significant correlations with plasma lipids, especially with the inverse correlation for HDL-cholesterol and positive correlations for LDL-cholesterol and triglycerides, suggest that salivary irisin measurements do indeed have physiologic significance.

The metabolic phenotype of PWS is unique. PWS individuals have abnormal body composition including decreased lean body and increased fat mass with less visceral adipose tissue and more subcutaneous fat tissue than is found in normal individuals with similar BMI [7,8]. Growth hormone deficiency and hypogonadism may contribute to the lower fasting insulin levels and greater insulin sensitivity in PWS compared to “normal” obese individuals [3,4,7]. Resting energy expenditure is reduced by about 16% in PWS adults [6]. As little as 50 g of stimulated BAT could account for 20% of daily energy expenditure in normal adults [9]. We considered the possibility that deficient irisin or irisin resistance in PWS might contribute to the need for severe caloric restriction in this syndrome and that abnormal secretion or regulation of irisin activity might contribute to some of the metabolic features of PWS.

Consistent with published reports from other investigators [3,4,7], our PWS patients had markedly lower fasting insulin, and less insulin resistance compared to non-PWS volunteers with comparable BMI. Fasting glucose in our PWS group was slightly lower than in controls, but hemoglobin A1c did not differ between the two groups. Fasting insulin and insulin resistance as measured by HOMA-IR were nearly 50% lower in the PWS group than in controls. Insulin sensitivity assessed by QUICKI was significantly greater in PWS than in controls. These differences were also significant when we compared overweight PWS patients to overweight controls. PWS patients with normal BMI, however, showed no difference in fasting glucose, insulin, or HOMA-IR compared to lean controls. Insulin sensitivity (QUICKI) was slightly, but significantly increased in lean PWS compared to lean controls.

Our data showing lower triglyceride levels in PWS compared to controls are consistent with previous studies in PWS children and adults [3,4]. In addition, we found that plasma irisin shown positive correlations with total cholesterol and LDL-cholesterol in PWS and in controls. Salivary irisin correlated inversely with HDL-cholesterol and positively with LDL-cholesterol and triglycerides in PWS, but not in normal controls. These correlations suggest that irisin might play a compensatory role in PWS to ameliorate the expected increased total and LDL-cholesterol and lower HDL-cholesterol levels in obese individuals. Previous reports found that plasma irisin correlates inversely with HDL-cholesterol in adults with the metabolic syndrome [19]. Others found that the decrease in circulating irisin following weight loss in obese adults correlated with the corresponding lowering of total and LDL-cholesterol [20]. Although it was suggested that irisin might have a role in regulating steroid synthesis [21,22], Wang *et al* found no effect of irisin on fatty acid metabolism in HepG2 hepatocytes [23].

Some investigators found that plasma irisin is higher in men, than in women [22], but we found no sex difference for levels of irisin in either PWS patients or controls. Another report described higher levels of irisin in young girls compared to boys [24]. It is possible that hypogonadism along with abnormal body composition, specifically low muscle mass, in PWS men might contribute to lower than normal levels of irisin and mask any gender effects on irisin levels.

Previous studies reported conflicting evidence regarding the effect of obesity on circulating irisin levels. Moreno-Navarette *et al* found that irisin levels were lower in obese and type 2 diabetic patients [25]. As in our study, others found no significant correlations between plasma irisin and BMI, blood glucose, or HbA1c [21,26–28]. Pardo *et al*, however, found higher irisin levels in obese individuals compared with lean controls [29].

Irisin has been shown to correlate with biceps circumference and lean body mass [22,30]. Since lean body mass is decreased in PWS, one might expect to find low levels of irisin in these patients. The fact that plasma irisin in our PWS patients was similar to levels in obese controls, suggests that circulating irisin in PWS may, in fact, be inappropriately high considering the abnormally low muscle mass in PWS. This relative “elevation” of plasma irisin in PWS may represent a compensatory mechanism or might indicate a state of irisin resistance. Interestingly, irisin levels did not differ in patients with sacropenia compared to controls with normal muscle mass [31].

The recent demonstration of immunoreactive irisin in the pancreatic islets of Langerhans, serous acini, and intralobular duct cells [32], raises the possibility that irisin might act not only as a circulating hormone, but might have autocrine or paracrine effects as well. Irisin secretion into the portal vein could affect insulin sensitivity by modulating insulin action in the liver. Pancreatic production of irisin might have local effects on enterocytes modulating lipoprotein metabolism. Since salivary glands share some common features with pancreatic function, salivary irisin might reflect production of irisin in the pancreas as well.

We measured plasma and salivary irisin on morning samples taken after an overnight fast in order to investigate correlations with insulin sensitivity and lipid profiles. We recognize that irisin samples taken after exercise might result in higher levels and perhaps show differences which were not apparent on the resting samples, however, it was not feasible in the framework of this study to require our PWS patients to exercise to a comparable degree as the control volunteers. Plasma irisin has been shown to exhibit diurnal variation, with peak levels observed at 9 pm [30]. Measurement of night-time irisin might demonstrate further differences which we did not find in the morning sample.

In a recent paper, Albrecht et al questioned the validity of current immunoassay methodologies for measuring circulating irisin [33]. Using four different commercial kits including an older version of the Phoenix antibody (EK-067-52), they found cross-reactivity with non-specific serum proteins. They did not, however, report results for the newer version of the Phoenix ELISA kit (catalogue number EK-067-29) which we used in our study. Mass spectroscopy findings by Lee et al [34], using the EK-067-29 kit showed that FNDC5 immunoreactive bands contain an irisin signature at 25 kDa and 32 kDa. The EK-067-29 antibody was also validated by Zhang et al [35] who visualise three bands (15, 20, and 26 kDa) on Western blot. The amino acid sequence of the molecules isolated from each of the three bands matched the amino acid sequence of irisin. The Phoenix ELISA kit has been used in most of the nearly 200 publications which showed significant correlations for immunoreactive irisin with physiologic and biochemical parameters in human and animal studies.

Abnormal regulation of irisin production might explain a nutritional phase observed in many PWS children, ages two to 4-1/2 years, during which they gain weight excessively without an increase in appetite or excessive calories [36]. Also, for reasons that are not understood, food-craving appears to diminish after the third or fourth decades of life [36]. The normal levels of irisin in our PWS adults might reflect the decreased food-craving seen in this age group. Further studies of irisin in PWS children and adults may contribute to understanding these phenomena.

This study, the first to report irisin levels in PWS, confirms previous findings showing greater insulin sensitivity, higher levels of leptin and adinopectin, and lower triglycerides in PWS adults compared with BMI-matched controls. Plasma irisin was associated with total and LDL-cholesterol in PWS patients and controls. Salivary irisin was markedly increased in PWS, and correlated with LDL-cholesterol and triglycerides, and inversely with HDL-cholesterol in PWS but not in control participants. Future studies are needed to determine whether irisin modulates insulin sensitivity and lipid profiles or, alternatively, if irisin levels are a reflection of the unique metabolic phenotype in PWS.

## Supporting Information

S1 Dataset Demographic and metabolic data. This supplementary file includes details of age, sex, genetic diagnosis for PWS, waist/hip ratios, BMI along with results of glucose, lipids, and hormone levels for each of the PWS and control participants in this study.
(XLSX)

S2 Dataset Saliva sodium and protein concentrations in PWS and controls. This supplementary file shows results of sodium and protein concentrations from five PWS individuals and for five control volunteers.
(XLSX)
